# The fNIRS glossary project: a consensus-based resource for functional near-infrared spectroscopy terminology

**DOI:** 10.1117/1.NPh.12.2.027801

**Published:** 2025-04-18

**Authors:** Katharina Stute, Louisa K. Gossé, Samuel Montero-Hernandez, Guy A. Perkins, Meryem A. Yücel, Simone Cutini, Turgut Durduran, Ann-Christine Ehlis, Marco Ferrari, Judit Gervain, Rickson C. Mesquita, Felipe Orihuela-Espina, Valentina Quaresima, Felix Scholkmann, Ilias Tachtsidis, Alessandro Torricelli, Heidrun Wabnitz, Arjun G. Yodh, Stefan A. Carp, Hamid Dehghani, Qianqian Fang, Sergio Fantini, Yoko Hoshi, Haijing Niu, Hellmuth Obrig, Franziska Klein, Christina Artemenko, Aahana Bajracharya, Beatrix Barth, Christian Bartkowski, Lénac Borot, Chiara Bulgarelli, David R. Busch, Malgorzata Chojak, Jason M. DeFreitas, Laura Diprossimo, Thomas Dresler, Aykut Eken, Mahmoud M. Elsherif, Lauren L. Emberson, Anna Exner, Talukdar Raian Ferdous, Abigail Fiske, Samuel H. Forbes, Jessica Gemignani, Christian Gerloff, Ségolène M. R. Guérin, Edgar Guevara, Antonia F. de C. Hamilton, S. M. Hadi Hosseini, Divya Jain, Anastasia N. Kerr-German, Haiyan Kong, Agnes Kroczek, Jason K. Longhurst, Michael Lührs, Rob J. MacLennan, David M. A. Mehler, Kimberly L. Meidenbauer, David Moreau, Murat C. Mutlu, Renato Orti, Ishara Paranawithana, Paola Pinti, Ali Rahimpour Jounghani, Vanessa Reindl, Nicholas A. Ross, Sara Sanchez-Alonso, Oliver Seidel-Marzi, Mohinish Shukla, Syed A. Usama, Musa Talati, Grégoire Vergotte, M. Atif Yaqub, Chia-Chuan Yu, Hanieh Zainodini

**Affiliations:** a Chemnitz University of Technology, Institute of Human Movement Science and Health, Faculty of Behavioural and Social Sciences, Chemnitz, Germany; b Birkbeck, University of London, School of Psychological Sciences, Faculty of Science, London, United Kingdom; c University of Birmingham, School of Computer Science, Birmingham, United Kingdom; d Boston University, Neurophotonics Center, Biomedical Engineering, Boston, Massachusetts, United States; e University of Padua, Department of Developmental Psychology and Socialisation, Padua, Italy; f ICFO-Institut de Ciències Fotòniques, The Barcelona Institute of Science and Technology, Barcelona, Spain; g Institució Catalana de Recerca i Estudis Avançats (ICREA), Barcelona, Spain; h University of Tuebingen, Tuebingen Center for Mental Health (TüCMH), Department of Psychiatry and Psychotherapy, Tuebingen, Germany; i University of L’Aquila, Department of Life, Health and Environmental Sciences, L’Aquila, Italy; j CNRS & Université Paris Cité, Integrative Neuroscience and Cognition Center, Paris, France; k University of Campinas, Institute of Physics, Campinas, Brazil; l University Hospital Zurich, University of Zurich, Biomedical Optics Research Laboratory, Department of Neonatology, Neurophotonics and Biosignal Processing Research Group, Zurich, Switzerland; m University of Bern, Institute of Complementary and Integrative Medicine, Bern, Switzerland; n University College London, Biomedical Optics Research Laboratory, Medical Physics and Biomedical Engineering, Faculty of Engineering, London, United Kingdom; o Politecnico di Milano, Dipartimento di Fisica, Milan, Italy; p Istituto di Fotonica e Nanotecnologie, Consiglio Nazionale delle Ricerche, Milan, Italy; q Physikalisch-Technische Bundesanstalt (PTB), Berlin, Germany; r University of Pennsylvania, Department of Physics and Astronomy, Philadelphia, Pennsylvania, United States; s Massachusetts General Hospital, Harvard Medical School, Athinoula A. Martinos Center for Biomedical Imaging, Charlestown, Massachusetts, United States; t Northeastern University, Department of Bioengineering, Boston, Massachusetts, United States; u Tufts University, Department of Biomedical Engineering, Science & Technology Center, Medford, Massachusetts, United States; v Hamamatsu University School of Medicine, Institute of Photonics Medicine, Division of Research and Development in Photonics Technology, Hamamatsu, Japan; w Beijing Normal University, State Key Lab of Cognitive Neuroscience and Learning, FNIRS Brain Imaging Group, Beijing, China; x Max Planck Institute for Human Cognitive and Brain Sciences, Leipzig, Germany; y University Hospital Leipzig, Faculty of Medicine, Clinic for Cognitive Neurology, Leipzig, Germany; z OFFIS—Institute for Information Technology, Health Department, Biomedical Devices and Systems Group, Oldenburg, Germany; aa RWTH Aachen University, Medical School, Department of Psychiatry, Psychotherapy and Psychosomatics, Aachen, Germany; ab University of Tuebingen, Department of Psychology, Faculty of Science, Tuebingen, Germany; ac Washington University School of Medicine, Mallinckrodt Institute of Radiology, St. Louis, Missouri, United States; ad Washington University in St. Louis, McKelvey School of Engineering, Imaging Science Program, St. Louis, Missouri, United States; ae German Center for Mental Health (DZPG), Tübingen, Germany; af University of Tuebingen, LEAD Graduate School & Research Network, Tuebingen, Germany; ag NIRx Medizintechnik GmbH, Berlin, Germany; ah TU Berlin, Medical Engineering, Faculty V—Mechanical Engineering and Transport Systems, Berlin, Germany; ai Liverpool John Moores University, School of Sport and Exercise Sciences, Faculty of Science, Liverpool, United Kingdom; aj University of Texas Southwestern, Departments of Anesthesiology and Pain Management, Neurology, Biomedical Engineering, Dallas, Texas, United States; ak University of Marie Curie-Sklodowska, Neuroeducation Research Lab, Faculty of Pedagogy, Lublin, Poland; al Syracuse University, Neural Health Research Laboratory, Syracuse, New York, United States; am Lancaster University, Department of Psychology, Faculty of Science and Technology, Lancaster, United Kingdom; an TOBB University of Economics and Technology, Department of Biomedical Engineering, Faculty of Engineering, Ankara, Turkey; ao University of Birmingham, Psychology, Birmingham, United Kingdom; ap University of British Columbia, Psychology Department, Vancouver, British Columbia, Canada; aq Ruhr University Bochum, Developmental Psychology, Faculty of Psychology, Bochum, Germany; ar University of Houston, Department of Biomedical Engineering, Houston, Texas, United States; as University of Oxford, Medical Sciences Division, Department of Experimental Psychology, Oxford, United Kingdom; at Durham University, Department of Psychology, Durham, United Kingdom; au Padova Neuroscience Center (PNC), Padua, Italy; av JARA Brain Institute II, Jülich Research Centre, Molecular Neuroscience and Neuroimaging (INM-11), Jülich, Germany; aw RWTH Aachen University, Child Neuropsychology Section, Department of Child and Adolescent Psychiatry, Psychosomatics and Psychotherapy, Aachen, Germany; ax University of Cambridge, Cambridge Centre for Data-Driven Discovery, Department of Applied Mathematics and Theoretical Physics, Cambridge, United Kingdom; ay Université Catholique de Louvain (UCLouvain), Institute of Neuroscience (IoNS), Brussels, Belgium; az CONAHCYT-Universidad Autónoma de San Luis Potosí, CIACYT, San Luis Potosí, Mexico, United States; ba University College London, Institute of Cognitive Neuroscience, London, United Kingdom; bb Stanford University, Computational Brain Research and Intervention (C-BRAIN) Lab, Department of Psychiatry and Behavioral Sciences, Palo Alto, California, United States; bc Icahn School of Medicine at Mount Sinai, Department of Rehabilitation and Human Performance, New York, United States; bd Mercer University, Psychology, Macon, Georgia, United States; be Nanjing Normal University, School of Psychology, Nanjing, China; bf Saint Louis University, Department of Physical Therapy and Athletic Training, St. Louis, Missouri, United States; bg Maastricht University, Department of Cognitive Neuroscience, Faculty of Psychology and Neuroscience, Maastricht, The Netherlands; bh Brain Innovation B.V., Research Department, Maastricht, The Netherlands; bi University of Florida, College of Medicine, Department of Neurology, Gainesville, Florida, United States; bj Malcom Randall VA Medical Center, Brain Rehabilitation Research Center, Gainesville, Florida, United States; bk University Hospital Münster, Institute for Translational Psychiatry, Medical Faculty, Münster, Germany; bl Cardiff University, College of Biomedical and Life Sciences, Cardiff University of Brain Research Imaging Centre (CUBRIC), Cardiff, United Kingdom; bm Washington State University, Department of Psychology, Pullman, Washington, United States; bn University of Auckland, School of Psychology, Auckland, New Zealand; bo University of Auckland, Centre for Brain Research, Auckland, New Zealand; bp Otto-von-Guericke University, Institute of Biology, Department of Cognitive Biology, Magdeburg, Germany; bq Otto-von-Guericke University, Center for Behavioral Brain Sciences, Magdeburg, Germany; br University of Campania Luigi Vanvitelli, Department of Psychology, Caserta, Italy; bs Monash University, Department of Electrical and Computer Systems Engineering, Faculty of Engineering, Clayton, Victoria, Australia; bt The Bionics Institute, East Melbourne, Victoria, Australia; bu Nanyang Technological University, School of Social Sciences, Department of Psychology, Singapore; bv University of Notre Dame, O’Sullivan Biophotonics Lab, Electrical Engineering, Notre Dame, Indiana, United States; bw Yale University, School of Medicine, Child Study Center, New Haven, Connecticut, United States; bx Friedrich Schiller University Jena, Department for the Psychology of Human Movement and Sport, Faculty of Social and Behavioural Sciences, Jena, Germany; by University of Amsterdam, Dept. of Psychology, Faculty of Social and Behavioral Sciences, Amsterdam, The Netherlands; bz Hörzentrum Oldenburg gGmbH, Oldenburg, Germany; ca Carl von Ossietzky University of Oldenburg, Department of Medical Physics and Acoustics, Oldenburg, Germany; cb Univ Montpellier, IMT Mines d’Alès, EuroMov Digital Health in Motion, Montpellier, France; cc University of Texas at Austin, Department of Kinesiology and Health Education, Austin, Texas, United States; cd University of Florida, College of Medicine, Department of Psychiatry, Gainesville, Florida, United States; ce Shahid Beheshti University of Medical Sciences, School of Medicine, Department of Biomedical Engineering and Medical Physics, Tehran, Iran

**Keywords:** functional near-infrared spectroscopy, glossary, continuous-wave near-infrared spectroscopy, time-domain near-infrared spectroscopy, frequency-domain near-infrared spectroscopy, diffuse correlation spectroscopy

## Abstract

**Significance:**

A shared understanding of terminology is essential for clear scientific communication and minimizing misconceptions. This is particularly challenging in rapidly expanding, interdisciplinary domains that utilize functional near-infrared spectroscopy (fNIRS), where researchers come from diverse backgrounds and apply their expertise in fields such as engineering, neuroscience, and psychology.

**Aim:**

The fNIRS Glossary Project was established to develop a community-sourced glossary covering key fNIRS terms, including those related to the continuous-wave (CW), frequency-domain (FD), and time-domain (TD) NIRS techniques.

**Approach:**

The glossary was collaboratively developed by a diverse group of 76 fNIRS researchers, representing a wide range of career stages (from PhD students to experts) and disciplines. This collaborative process, structured across five phases, ensured the glossary’s depth and comprehensiveness.

**Results:**

The glossary features over 300 terms categorized into six key domains: analysis, experimental design, hardware, neuroscience, mathematics, and physics. It also includes abbreviations, symbols, synonyms, references, alternative definitions, and figures where relevant.

**Conclusions:**

The fNIRS glossary provides a community-sourced resource that facilitates education and effective scientific communication within the fNIRS community and related fields. By lowering barriers to learning and engaging with fNIRS, the glossary is poised to benefit a broad spectrum of researchers, including those with limited access to educational resources.

## 1 Motivation

With the advent of wearable neuroimaging technology, the applications and number of users of functional near-infrared spectroscopy (fNIRS) are rapidly growing.^1^^–^^5^ The use of fNIRS has led to advances in different fields such as cognitive neuroscience,^6^^,^^7^ clinical applications,^8^^–^^10^ developmental science,^11^^,^^12^ and sport and exercise science.^13^^,^^14^ Recent community-based efforts have played a key role in establishing fNIRS as a neuroimaging technology. Examples of these community-based efforts include a consensus paper with guidelines to help enhance the reliability, repeatability, and traceability of reported fNIRS studies^2^ and a guide and template on using preregistration as a tool for transparent fNIRS study design^15^ as part of adopting open science practices.^16^ Another example is the creation of a standardized file format, namely the shared near-infrared spectroscopy file format (.snirf), to facilitate the analysis and sharing of fNIRS data,^17^ and the adoption of the Brain Imaging Data Structure (BIDS) that specifies how data sets should be organized to facilitate the sharing of data sets across research labs or from different manufacturers to further enhance reproducibility of fNIRS studies.^18^ Continuing the desire for standardization in fNIRS, the FRESH (fNIRS REproducibility Study Hub) project^19^ explores the impact of analytical flexibility on research conclusions drawn when independent fNIRS researchers analyze the same dataset (see Ref. 20).

Despite efforts to enhance reproducibility and promote standardization, the growing body of knowledge in fNIRS continues to widen the knowledge gap and steepen the learning curve, making it challenging for new users to enter the field. This gap is particularly pronounced because fNIRS intersects with various complex disciplines such as physiology, cognitive neuroscience, hardware engineering, and physics, demanding a minimum level of expertise in several fields from beginners. For example, the word “probe” in a general context is a verb, which means to investigate something, and in a medical imaging context, it usually denotes a physically invasive measurement, such as an intracranial pressure probe. However, in the context of fNIRS, “probe” refers to a light source or light detector “probe,” which is neither a verb nor invasive. There are also instances where many different terms are used that have practically similar meanings, for example “source,” “light source,” “emitter,” and “transmitter,” which in this case all mean a source of light. The fNIRS Glossary Project also provides a synonyms field on the online version which contains the synonyms for terms where synonyms are commonly used.

The fNIRS Glossary Project, with the motivation to tackle these challenges, built a community-sourced glossary (i.e., useful nomenclature and related symbols) of terms related to fNIRS technologies, covering the experimental design, pre-processing, data analysis, neuroscience, physics, and mathematical areas that are crucial to understanding and using fNIRS successfully and reliably in research, industry, and clinical applications. Herewith, the glossary will serve to clarify terminologies to aid new researchers entering the field and experts to communicate efficiently through a common understanding and usage of terminologies.

With this project, we offer the first glossary for the fNIRS community, similar to the work in the field of EEG^21^ and cognitive neuroscience.^22^ This glossary goes beyond reporting to cover all stages of the research process—from experimental design to analysis—and includes essential terms related to technical and experimental aspects of the field, helping researchers better understand the technology. We envisage that the glossary will further lower the barrier to follow and join discussions and thus also help to enhance diversity, equity, and inclusion in the rapidly expanding field of fNIRS research.^2^

## 2 Methods: The fNIRS Glossary Project

**Fig. 1:**
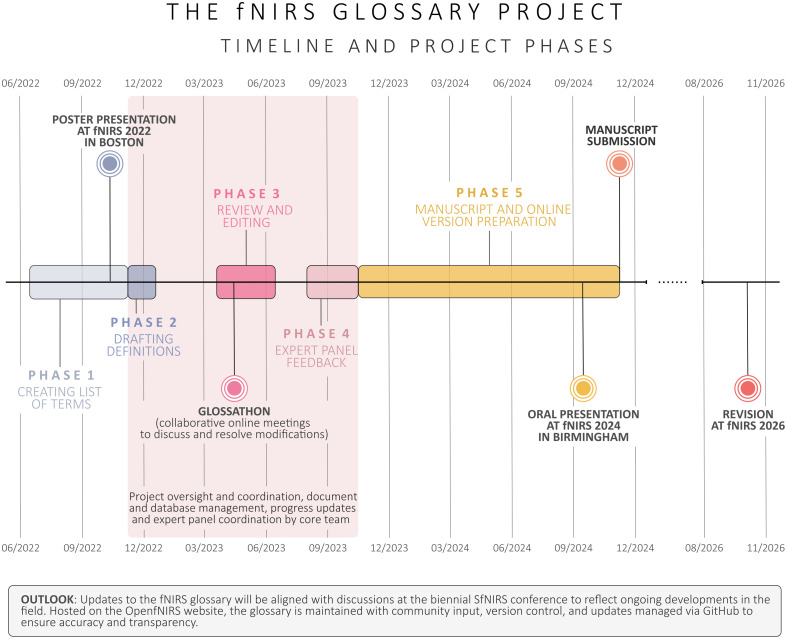
Timeline and milestones of the project.

The fNIRS Glossary Project was carried out over five main phases. Figure 1 illustrates the timeline of the different project phases and the key milestones.

### 2.1 Recruitment and Characteristics of Community Members

The fNIRS Glossary Project has been collaboratively developed by a diverse group of 76 fNIRS researchers from various career stages (from students and early career researchers to longstanding experts in the field) and disciplines (such as biomedical engineering, physics, psychology, medicine, neuroscience, sport and exercise science amongst others). The fNIRS Glossary Project was presented as a poster during the bi-annual conference of the Society for Functional Near-Infrared Spectroscopy (SfNIRS) in Boston, in October 2022, to introduce the project to the community and to gather collaborators. We disseminated a Google sign-up form, allowing researchers to express their interest in joining the project and receive future updates. In addition, the project was disseminated across various social media platforms, including the official Facebook group of the SfNIRS, a project page on ResearchGate, X (formerly Twitter) as well as invitations extended personally to colleagues.

### 2.2 Phase 1: Creating the List of Terms

In the first phase (June 15, 2022, to November 7, 2022), the fNIRS Glossary Project lead team (KS, LKG, SMH, GAP, and MAYüc) compiled an initial list of terms related to fNIRS, its technology including continuous-wave (CW), frequency-domain (FD), and time-domain (TD) NIRS techniques, hardware, data analysis, and experimental design, as well as background information on neuroscience and physics terms that are crucial to understanding fNIRS and how the technology works. This initial list consisted of N=324 terms, with n=112 drawn from the OntoNIRS project,^23^ an ontology for the automatization of fNIRS experimental design and analysis, n=18 from the International Electrotechnical Commission (IEC)/International Organization for Standardization (ISO) standard for fNIRS equipment (IEC 80601-2-71:2015),^24^ and n=5 from the ISO/IEC standard for cerebral tissue oximeters (ISO 80601-2-85:2021).^25^

### 2.3 Phase 2: Drafting Definitions

In the second phase (November 8, 2022, to December 18, 2022), researchers who expressed interest in joining the project through the Google sign-up form were invited to select three terms from the initial list or propose new ones. For each term, contributors were required to provide a definition, a category (i.e., analysis, experimental design, hardware, mathematics, neuroscience, physics), related terms, abbreviations or symbols, synonyms, references, and an alternative definition (if applicable). Submissions were collected through a structured Google form.

### 2.4 Phase 3: Review and Editing

In the third phase (March 20, 2023, to June 15, 2023), all submitted entries were divided into different documents sorted by category (that is, analysis, experimental design, hardware, maths, neuroscience, physics). Furthermore, all terms were assigned a status field: *Open for Review*, *Pending* (pending at least five team members’ approval), *Agreement* (agreement by at least five team members of the respective category), *In-Conflict* (requires expert panel review), or *No Review* (for terms taken from IEC/ISO standards). The categories were primarily monitored by a lead team member (Analysis: MAYüc, Experimental design: KS, Hardware: SMH, Maths: GAP, Neuroscience: LKG, Physics: GAP). All members of the glossary team were asked to join the category according to their main area of expertise, with the option to choose more than one category. They were required to work on at least 15 terms (revising/reviewing the definitions drafted in the previous phase). In this phase, we organized and hosted collaborative online meetings, referred to hereafter as Glossathons, for each main category to collectively discuss and resolve modifications. These meetings took place from April 17, 2023, to April 21, 2023. At least five team members had to agree on a term to mark it as a completed definition. The status field was overseen and managed by the lead team members responsible for the respective category.

### 2.5 Phase 4: Expert Panel Feedback

In the fourth phase (July 31, 2023, to October 15, 2023), we invited a group of expert fNIRS users some of which had already contributed to phase 1 (see Sec. 2.2) and phase 2 (see Sec. 2.3) of the fNIRS Glossary Project (SC, TDur, ACE, JGev, RCM, FOE, VQ, FS, AT, HW) and some which were new to the project (SAC, HD, QF, SF, MF, HN, HO, IT, AGY) to review the category documents to further improve the definitions and resolve terms that had been marked as in-conflict in phase 2. Experts were fNIRS researchers with considerable experience in the field (15+ years). We aimed to include experts with diverse backgrounds. Each category document was reviewed by the following expert panel members: analysis (RCM, FOE, FS, IT, HD, QF, HN), experimental design (SC, ACE, JGev, FS), hardware (TDur, IT, AT, HW, SAC), Maths (RCM, FOE), neuroscience (SC, ACE, MF, JGev, VQ, IT, YH, HO), and physics (TDur, MF, RCM, VQ, IT, AT, HW, AGY, SAC, SF, YH). Experts resolved n=6 in-conflict terms (analysis: n=1; experimental design: n=1; neuroscience: n=4) and provided input on terms that were especially important to the fNIRS field.

### 2.6 Phase 5: Manuscript and Online Version Preparation

In the fifth phase (October 16, 2023, to November 16, 2024), the fNIRS Glossary Project lead team integrated the expert panel feedback, added additional reviewers to terms where needed, and prepared the manuscript and the online version for the final journal submission. All team members and expert panel reviewers were invited as co-authors on this manuscript. The project resulted in the drafting of N=322 terms (analysis: n=71, experimental design: n=42, hardware: n=53, Maths: n=12, neuroscience: n=77, physics: n=67). Further, we included four terms that were already defined in the IEC/ISO standards for fNIRS equipment (IEC 80601-2-71)^24^ and cerebral tissue oximeters (ISO 80601-2-85).^25^ In Sec. 5, we present an excerpt of the glossary of 32 key terms. The finalized fNIRS Glossary, including references and links to related terms, is available at Ref. 26, where a PDF version can also be downloaded. The fNIRS Glossary Project is licensed under a CC BY NC SA 4.0 license. The current glossary is the version 1. We documented the contributions of all team members using the Contributor Roles Taxonomy (CRediT).^27^

### 2.7 Challenges

We consider it vital to outline the challenges that we encountered and solved during this community-based project so that future projects such as this one may learn from our experience. A key challenge for the project was to ensure that all essential and topic-specific fNIRS terms were included in the compiled list for phase 2, without making the selection too broad. The initial list was compiled by the lead team, drawing on terms from the OntoNIRS project.^23^ We ensured a comprehensive list of key terms by allowing multiple rounds of additions as the number of team members grew. Subsequently, glossary project members and, in a final revision, domain experts had the opportunity to contribute new terms. We are therefore confident that we have covered the most essential terms. By hosting our project on the openfNIRS website (see Ref. 26), we maintain the flexibility to add new terms in future iterations. Those who wish to suggest a new term can do so via a Google form linked on the webpage.

The second and most important challenge to address was the content of the definitions. Definitions needed to be (1) comprehensible to all levels of fNIRS users, even novice users, and (2) complete and detailed, to also be of use to experienced fNIRS users. We ensured this by allowing junior researchers to contribute to the glossary project at all stages of the project. To ensure the quality of the term definitions, consensus from at least five fNIRS community members and approval from an expert panel member were required for each term. Each document lead (i.e., analysis: MAYüc, experimental design: KS, hardware: SMH, maths: GAP, neuroscience: LKG, physics: GAP) then also double-checked the terms. A Glossathon that took place in April 2023 also served as a good way to push towards consensus definitions.

## 3 Results

**Fig. 2:**
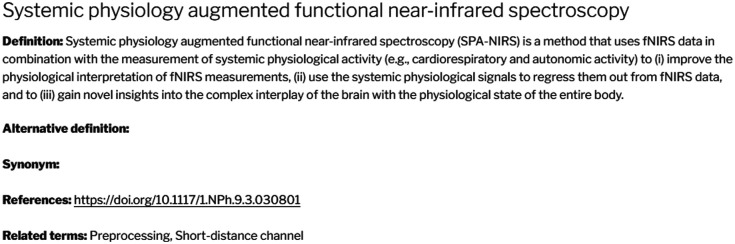
Example of a term entry in the fNIRS Glossary Project online version, displaying all available fields.

The final fNIRS glossary document can be found under Ref. 26. In the online glossary, each term appears in the form that can be seen below (see Fig. 2), with the fields of definition, alternative definition, synonym, references, and related terms. The Appendix contains example key terms and their definitions to illustrate the format of the glossary, which are sampled from all categories.

## 4 Discussion

This project serves as a resource for newcomers and experts in the field of fNIRS by providing the definitions of fNIRS-related terms and bridging the communication gap across disciplines. While making recommendations on preferred acronyms for fNIRS technology (e.g., for oxygenated hemoglobin) is beyond the scope of this project, we aim to update the glossary once a consensus on acronyms has been reached within the field. Given the rapid developments in the field, we anticipate that terms will continue to evolve, requiring regular updates and revisions.

Hosting the project on the openfNIRS website (see Ref. 26) ensures consistency in terminology display, facilitates maintenance, and allows seamless integration of new terms in future revisions. Community members have the opportunity to submit new terms for consideration via a Google form linked on the website. These submissions will be reviewed by the fNIRS Glossary lead team, in collaboration with the SfNIRS community. Discussions on updates will take place during the biennial SfNIRS conference, and agreed-upon changes will then be incorporated into the glossary.

To ensure transparency and accuracy, future updates will use GitHub to incorporate version control to track corrections and the addition of newly defined terms and references. The first version of the glossary was compiled in CSV format and is publicly available on GitHub at https://github.com/fnirsglossaryproject/glossary. The revisions will be managed through this repository by the fNIRS Glossary lead team.

Recognizing the value of accessibility for non-native English speakers, we are also exploring multilingual translations of the glossary (e.g., Spanish, Chinese) to support those new to fNIRS.

## 5 Appendix: Example Terms from the fNIRS Glossary

### 5.1 Absorption of Light

Absorption is the transformation of the photon energy from electromagnetic radiation (such as visible or near-infrared light) into another form of internal energy by an atom or a molecule. In an absorbing medium, absorption reduces the amount of light as the light passes through the medium.

### 5.2 Block Design

**Fig. 3:**
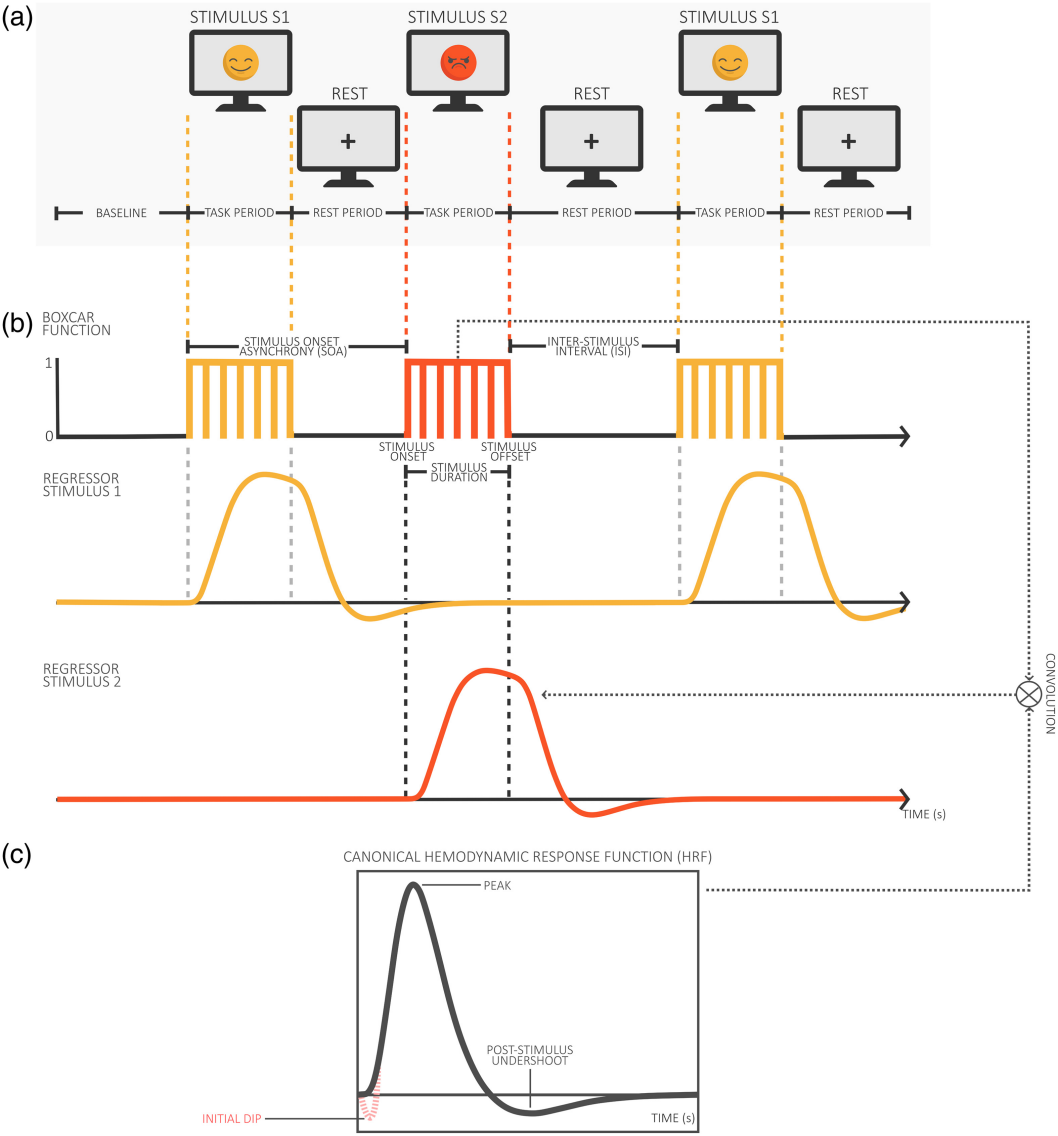
Graphical representation of a block design illustrating task and rest periods, with key terms such as stimulus onset asynchrony (SOA), interstimulus interval (ISI), and the convoluted hemodynamic response function (HRF).

A block design (Fig. 3) is a type of experimental paradigm in which trials belonging to the same experimental condition are arranged in blocks (i.e., grouped together), with blocks usually separated by a rest period. The original purpose of a block design is to increase the signal-to-noise ratio (SNR) and to reduce the effects of variability within individual trials.

### 5.3 Broadband NIRS

Broadband NIRS (bNIRS) is an optical imaging technique that measures *in vivo* changes in tissue hemodynamics (oxyhemoglobin and deoxyhemoglobin) and, often, other biomarkers such as metabolism (e.g., redox state of cytochrome-c-oxidase or (oxCCO)). Tissue light attenuation measurements are performed at a larger number of wavelengths than typical two- to four-wavelength NIRS instruments, e.g., spanning 600 to 1000 nm at 1 nm wavelength resolution, to reduce crosstalk effects and noise.

In contrast to fNIRS systems, bNIRS use broadband light sources, for example, a tungsten halogen light source-emitting light between 200 and 2500 nm and a wavelength-dependent detector (e.g., a spectrophotometer). Optical filters may be placed in the source or detector optical path to narrow the spectrum to the region of interest.

### 5.4 Channel

**Fig. 4:**
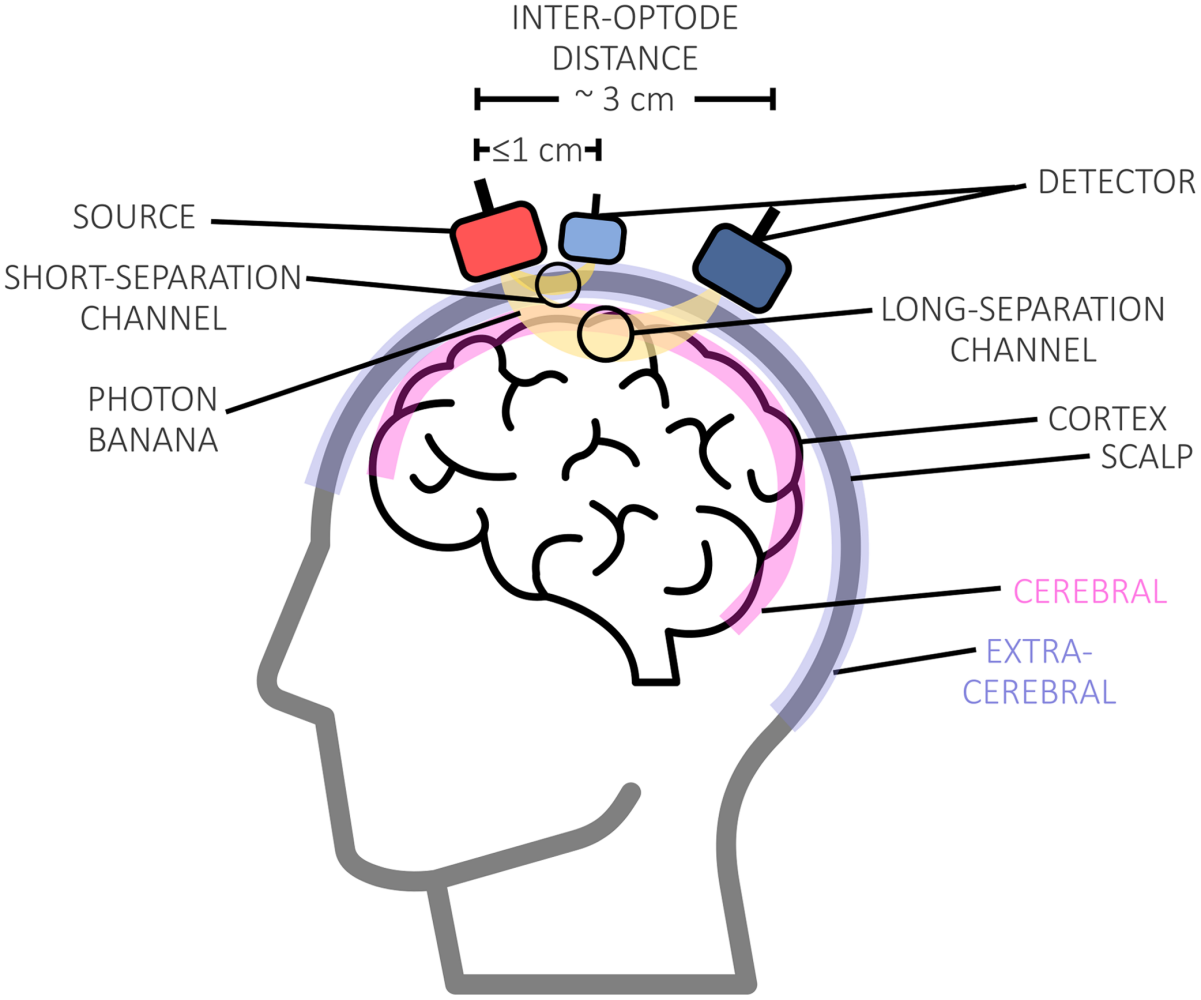
Graphical representation of a typical fNIRS measurement setup, highlighting key terms such as the source and detector, which form a channel, as well as short- and long-separation channels, among others.

A channel (Fig. 4) is a measurement point that the system is capable of recording. In the special case of fNIRS, a channel is defined as the area between a light source and a detector and is often visually depicted as the midpoint of the two and at a depth of approximately half of the source-detector distance. The source-detector distance categorizes a channel as a typical long-separation fNIRS channel (reflecting cortical activation changes and changes in systemic physiology) or a short-separation/short-distance channel (primarily reflecting changes in skin perfusion).

### 5.5 Chromophore

A chromophore is a molecule that absorbs light within a specific wavelength range, spanning from ultraviolet to infrared light. Within the near-infrared spectrum, the primary chromophores in biological tissues include water, hemoglobin, lipids, and cytochrome oxidase. Notably, light achieves its deepest penetration into tissue at wavelengths approximately between 700 and 900 nm. Functional near-infrared spectroscopy (fNIRS) employs multiple wavelengths to sensitively distinguish between changes in the concentrations of oxyhemoglobin and deoxyhemoglobin, providing valuable insights into tissue oxygenation and, by extension, neural activity.

### 5.6 Continuous-Wave Spectroscopy

Continuous wave (CW) spectroscopy is an fNIRS technique in which light of constant intensity is injected into tissue, and then, the attenuated light signal is measured at a distance from the light source. Note, in practice, all sources are switched off/on on some timescale; when the on/off time is long compared with relaxation/equilibrium in the sample, then such sources are considered to be continuous from the physics point of view.

### 5.7 Cross-talk

By cross-talk, we mean to describe the phenomenon that a genuine change in one chromophore concentration is also inducing a spurious measured concentration change in another. These phenomena can also apply to cross-talk between changes in the absorption and scattering coefficient.

In the context of the hardware, cross-talk can also be applied in the context of the physical measurement of light, where a direct measurement between a single source and detector can be contaminated by other sources of light. This is usually mitigated by the use of time multiplexing or frequency multiplexing. When utilizing silicon photomultipliers for photodetection, optical cross-talk may occur when an incident photon hits a microcell. This initial discharge can cause additional discharges in adjacent microcells, leading to an undesired amplification of the signal output.

### 5.8 Depth Sensitivity

In the context of fNIRS, which typically uses an array of measurement optodes on the scalp to measure brain activity, depth sensitivity refers to its ability to detect localized changes in deep tissue optical properties. “Depth” is typically measured normal to the surface of the skin. Sensitivity to depth depends on multiple factors in the instrumentation (e.g., illumination strength, detector efficiency, source-detector separation, data type) and tissue optical properties (e.g., chromophore concentration). It is crucial to note here that there is no singular depth being probed by fNIRS, but rather, it has a continuous distribution over the probed tissue volume which can change with time and with optical properties. Typically, the longer the distance between the source and detector across the surface of the measurement body (scalp, phantom, etc.), the deeper the depth sensitivity.

### 5.9 Differential Path Length Factor

The differential path length factor (DPF) is a unitless factor that accounts for the differential pathlength of photon travel in human tissue. The DPF is calculated by dividing the mean optical pathlength by the distance between the emitter and detector. The DPF depends on age, gender, and wavelength.

### 5.10 Diffuse Correlation Spectroscopy

Diffuse correlation spectroscopy (DCS) uses the temporal fluctuations of speckles formed on the tissue surface by coherent light to derive metrics for the motion of scatterers in highly scattering media such as blood cells flowing in tissue vasculature. The DCS technique was formulated based on the correlation diffusion equation; it is formally equivalent to the technique (formulated as an integral equation rather than a differential equation) called diffuse wave spectroscopy (DWS). In the diffuse optics community, the term DWS sometimes implied absorption spectroscopy; hence, the term DCS was introduced to reduce terminology confusion. The word “correlation” in the name is connected with the fact that canonical DCS uses the auto-correlation function of the intensity fluctuations as its primary measured quantity.

Apart from the usual optical properties (absorption and reduced scattering coefficients), the DCS signal (decay of the measured curve) in tissue is dominated by the movement of red blood cells which permits DCS to estimate a blood flow index. Standard DCS measures the autocorrelation function and fits these measurements to solutions of the correlation diffusion equation for the appropriate geometry and boundary conditions. DCS is closely related to the other speckle correlation techniques such as interferometric DCS and DWS (iDCS/iDWS), speckle contrast optical spectroscopy and tomography (SCOS/SCOT), laser speckle contrast imaging (LSCI), and diffuse correlation tomography (DCT). Other implementations of DCS have been described in the literature.

### 5.11 Frequency-Domain Near-Infrared Spectroscopy

Frequency-domain near-infrared spectroscopy (FD-NIRS) is an fNIRS technique that sinusoidally modulates a near-infrared light source at a given frequency into the tissue and detects the phase shift as well as the attenuation in amplitude with the aim of estimating the absorption and scattering coefficients of the medium. The modulation frequency needs to be higher than the shift induced by the time of light propagation inside the medium (on the order of nanoseconds), which depends on the light speed in the medium. Usually, this is on the order of 100 MHz. FD spectroscopy can vary the modulation frequency (multi-frequency FD) and measure the amplitude attenuation and phase shift at different frequencies. In some cases, it is possible to measure attenuation and phase at one single frequency and use modeling assumptions to estimate the absorption and scattering coefficients and in turn quantify chromophore concentrations.

### 5.12 Functional Near-Infrared Spectroscopy

Functional near-infrared spectroscopy (fNIRS) is a non-invasive diffuse optical technique that quantifies changes in measured signals from a single or several source-detector pairs over short periods of time (ranging from seconds to hours) to detect functional brain activation. Typically, it is assumed that the changes in light intensity during this temporal range reflect changes in absorption only (i.e., scattering is constant during this period). The changes in absorption can then be related to changes in chromophore concentration such as oxy- and deoxy-hemoglobin, water, and cytochrome c oxidase. fNIRS is performed with continuous-wave spectroscopy devices, as well as frequency-domain and time-domain spectroscopy techniques. In the case of frequency-domain and time-domain systems, it is possible to obtain absolute parameters of optical properties and therefore absolute concentration changes, but this is less commonly performed.

fNIRS measurements with multiple measurement channels may use image reconstruction methods, which when done for 2D (topographic) or 3D (tomographic) images fall into the domain of diffuse optical imaging.

### 5.13 Hemodynamic Response

The hemodynamic response is a change in blood flow in response to an increase in local metabolic demands due to an increase in neuronal activity. In the measured fNIRS signal, this is reflected as a change in oxy- and deoxy-hemoglobin concentrations. Typically, the blood flow response over-compensates the demand leading to a focal hyperoxygenation in the activated area. The peak of the hemodynamic response is detectable by NIRS at about 6 s (4 to 8 s) after the onset of the event. The hemodynamic response is typically characterized by a large increase in oxy-Hb and a smaller decrease in the concentration of deoxygenated hemoglobin (but may—depending on the measurement area—also show no change or even increase during a hemodynamic response) resulting in the characteristic anticorrelated signal structure of oxy- and deoxy-hemoglobin. Features of the hemodynamic response function (HRF) have been shown to change with age or disease.

### 5.14 Hyperscanning

The simultaneous recording of brain activity from more than one person. Typically, a hyperscanning setup is used when the relationship between the participants’ brain activations is of interest; thus, during hyperscanning studies, the participants are able to communicate with one another either verbally or non-verbally, for example, by being in the same room or by seeing each other’s actions.

### 5.15 Image Reconstruction

Image reconstruction in functional near-infrared spectroscopy (fNIRS) is the process of generating 2D or 3D spatially varying images of the hemodynamic response in the brain using recorded signals from fNIRS optodes on the scalp, typically using a head model as a spatial prior (either subject-specific or atlas-based), often referred to as diffuse optical tomography (DOT). This is an approach for image reconstruction in fNIRS, which uses a computational model of light propagation within the head (forward problem) to estimate the distribution of hemodynamic changes that give rise to the measured signal (inverse problem). The forward problem and inverse problem are two important steps in image reconstruction. The forward problem refers to the calculation of the distribution of light propagation (intensity, phase, time of flight) in the head, given the distribution of the light sources and detectors on the scalp while the inverse problem refers to the estimation of the hemodynamic response in the brain tissue from the measured signal.

### 5.16 Modified Beer–Lambert Law

A common approach to measuring changes in tissue absorption using continuous wave methods is based on a generalization of Beer–Lambert law in conjunction with the assumption that the scattering properties of tissue do not vary with time. The modified Beer–Lambert law (mBLL) can be expressed as follows [Eq. (1)]:

$$I=I_{0}e^{(-\overset{¯}{\mathrm{DPF}}\mu_{a}r)-G},$$ (1)

where I is the detected optical intensity. $I_{0}$ is the incident intensity. $\bar{\mathrm{DPF}}$ is an average (over the absorption range from 0 to $\mu_{a}$) mean pathlength factor that depends on the optical properties of tissue, $\bar{\mathrm{DPF}}$ accounts for the dependence of the mean optical pathlength on scattering and absorption, and r is the inter-optode distance. G is a factor that accounts for the effect of scattering, and $\mu_{a}$ is the absorption coefficient.

By calculating the logarithmic ratio of I and $I_{0}$, we estimate the attenuation OD (optical density) [Eq. (2)]:

$$A=\mathrm{OD}=\log_{10}\;\frac{I_{0}}{I}=\frac{\overset{¯}{\mathrm{DPF}}\mu_{a}r+G}{\log_{e}(10)},$$ (2)

which is the integral form of the mBLL. It is valid for an arbitrary medium in terms of its geometry and spatial distribution of the scattering coefficient.

If we assume that G is constant and that the absorption change in the medium between a baseline condition and a test condition is small compared with $\mu_{a}$, it can be shown that the logarithmic difference between the detected intensity at baseline and during the test condition can be written as follows [Eq. (3)]:

$$\Delta A=\Delta \mathrm{OD}=\log_{10}(\frac{I_{\text{baseline}}}{I_{\text{test}}})=\mathrm{DPF}\frac{r\Delta \mu_{a}}{\log_{e}(10)},$$ (3)

where the differential pathlength factor (DPF) is defined as the ratio of the mean pathlength of detected photons at baseline to the inter-optode distance. The last equation is the differential form of the mBLL, and it is valid for an arbitrary medium in terms of its geometry and spatial distribution of the optical properties at baseline. The only requirement is that $\Delta \mu_{a}$ is spatially uniform.

The change in the absorption coefficient $\Delta \mu_{a}$ is the summation of the changes in the absorption contributions associated with available chromophores in the medium, e.g., ${\mathrm{HbO}}_{2}$ and Hb which are given by the product of their molar decadic absorption coefficients $\left(\epsilon_{{\mathrm{HbO}}_{2}},\epsilon_{\mathrm{Hb}}\right)$ and their concentration changes [Eq. (4)]:

$$\Delta \mu_{a}(\lambda )=\log_{e}(10)\cdot [\epsilon_{{\mathrm{HbO}}_{2}}(\lambda )\Delta c_{{\mathrm{HbO}}_{2}}+\epsilon_{\mathrm{Hb}}(\lambda )\Delta c_{\mathrm{Hb}}].$$ (4)

The change in absorbance can then be written for two wavelengths ($\lambda_{1}$ [Eq. (5)], $\lambda_{2}$ [Eq. (6)] and two chromophores (${\mathrm{HbO}}_{2}$, Hb) as below, assuming DPF to be independent of wavelength:

$$\Delta A(\lambda_{1})=\mathrm{DPF}\cdot r\cdot [\epsilon_{{\mathrm{HbO}}_{2}}(\lambda_{1})\Delta c_{{\mathrm{HbO}}_{2}}+\epsilon_{\mathrm{Hb}}(\lambda_{1})\Delta c_{\mathrm{Hb}}],$$ (5)

$$\Delta A(\lambda_{2})=\mathrm{DPF}\cdot r\cdot [\epsilon_{{\mathrm{HbO}}_{2}}(\lambda_{2})\Delta c_{{\mathrm{HbO}}_{2}}+\epsilon_{\mathrm{Hb}}(\lambda_{2})\Delta c_{\mathrm{Hb}}].$$ (6)

One can estimate the concentration changes of both hemoglobin species by solving this system of linear equations.

### 5.17 Molar Absorption Coefficient

The molar (decadic) absorption coefficient (formerly: molar decadic extinction coefficient) is an intrinsic property of a given molecule (chromophore) or substance that describes how strongly that molecule/substance absorbs light at a particular wavelength. It is equal to absorbance (A, base 10) divided by the absorption pathlength, d, and the amount concentration, c [Eq. (7)]

$$\epsilon (\lambda )=-\frac{1}{cd}\;\log_{10}(\frac{I_{d}}{I_{0}})=\frac{A(\lambda )}{cd}.$$ (7)

It is generally represented by the unit $M^{-1}\;{\mathrm{cm}}^{-1}(={\mathrm{Lmol}}^{-1}\;{\mathrm{cm}}^{-1})$.

### 5.18 Motion Artifact

**Fig. 5:**
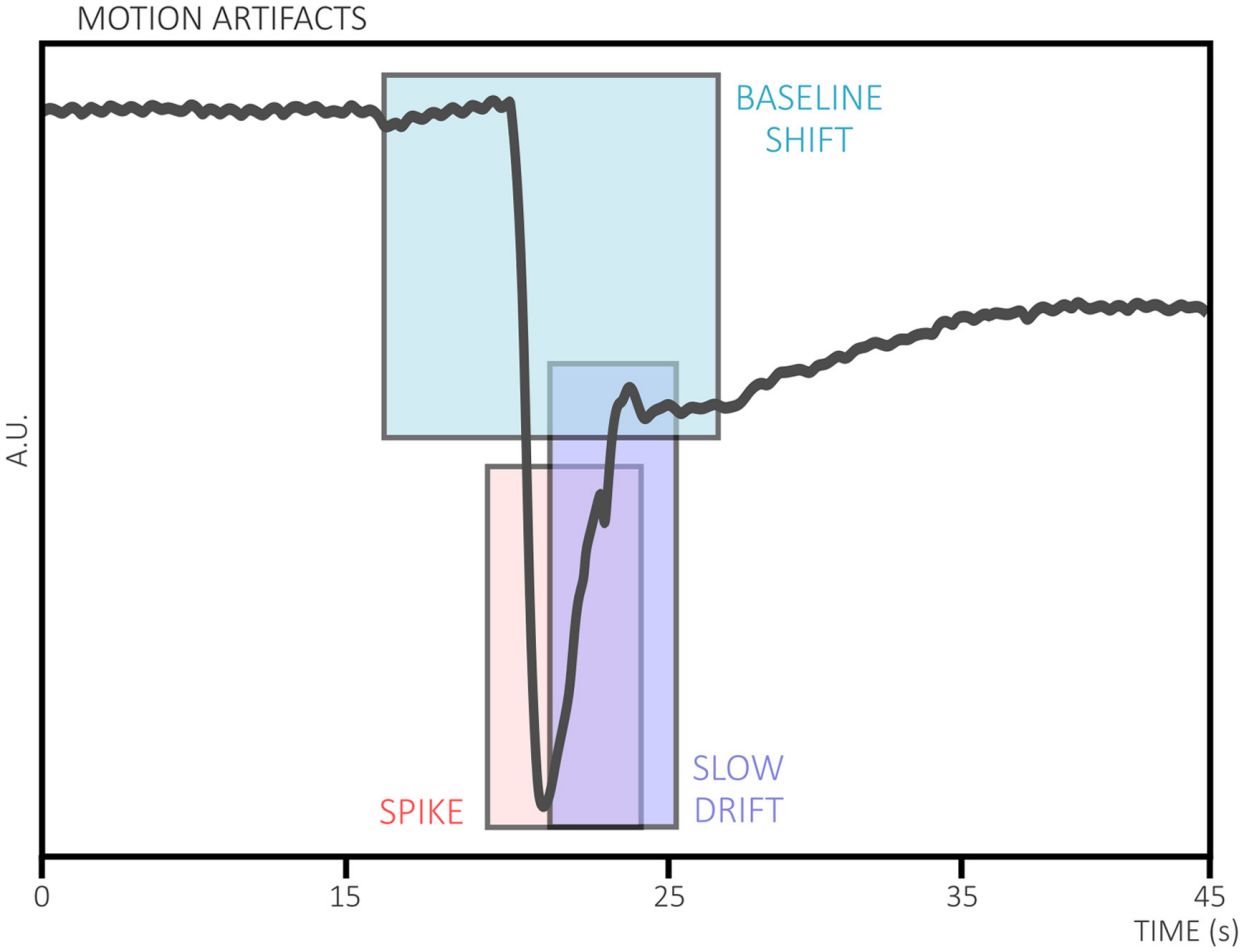
Different common types of motion artifacts in fNIRS research.

Motion artifacts (Fig. 5) are changes in the signal caused by the movements of the subject being measured. In the context of fNIRS, one major cause of motion artifacts is the scalp-optode decoupling due to head or skin movement. Such a decoupling can cause high-frequency spikes in the signal if the optode recouples with the scalp at the same location, or it can create baseline shifts in the signal if the optode settles on a different location on the scalp after the motion. Motion artifacts can significantly reduce the quality of the fNIRS data making it challenging to estimate underlying hemodynamic changes.

### 5.19 Optical Density

Optical density (or absorbance) is defined as the (decadic) logarithm of the ratio of incident to transmitted radiant power through a (non-scattering) sample. This quantity is used, e.g., in spectrophotometry to characterize a dissolved chromophore in a non-scattering solution or an optical filter. Absorbance is defined as [Eq. (8)]

$$A=-\log_{10}\;T=-\log_{10}(\frac{I_{d}}{I_{0}}),$$ (8)

where A is the absorbance or optical density, T is the transmittance, $I_{d}$ is the intensity of the transmitted light after the light traveling a pathlength d, and $I_{0}$ is the intensity of the incident light.

For a single chromophore, T can be written as [Eq. (9)]

$$T=e^{-\mu_{a}d}=10^{-\epsilon cd},$$ (9)

where a $\mu_{a}$ is the absorption coefficient, ϵ is the molar decadic absorption coefficient, and c is the concentration of the chromophore. This leads to Eq. (10):

$$A=\epsilon cd=\frac{\mu_{a}d}{\log_{e}\;10},$$ (10)

where $\mu_{a}$ is given by Eq. (11) and it should be noted that $\log_{e}\;10=2.30258\ldots \approx 2.303$,

$$\mu_{a}=\log_{e}\;10\cdot \epsilon c=2.303\cdot \epsilon c.$$ (11)

In fNIRS, optical density (OD) is sometimes used as a synonym for (decadic) attenuation (termed A as well). In this context, attenuation is relevant instead of absorbance because it also includes the effect of light scattering.

### 5.20 Oxygenated Hemoglobin

Oxygenated hemoglobin is a complex molecule where the heme group of the hemoglobin protein has formed a reversible complex with oxygen ($O_{2}$) in the pulmonary capillaries. This red blood cell protein travels through circulating blood carrying oxygen to body tissues and organs where it is readily released.

### 5.21 Partial Volume Effect

The partial volume effect can be defined as the loss of contrast in small objects or regions due to the limited resolution of the imaging system. Thus, it refers to the underestimation of the concentration changes of hemoglobin due to the fact that they occur in small focal regions rather than in the entire sampling region.

### 5.22 Physiological Noise

In the context of fNIRS, physiological noise reflects physiological activity (e.g., blood pressure fluctuations related to heart rate, respiration, and low-frequency oscillations) that are not related to the hemodynamic signal of interest, i.e., the hemodynamic signal that indexes neural activity. Physiological noise can be captured as a global component in overall brain activity, or measured in surface vasculature, later to be removed from the fNIRS signal.

### 5.23 Probe

A probe or probeset is the arrangement of optodes on the head. Probes can be placed according to the international 10 to 20 system and/or other anatomical landmarks. The optodes (emitters and detectors) are typically located in a grid with fixed inter-optode distances for long-distance and short-distance channels.

### 5.24 Registration

Registration is the process of spatially registering, i.e., matching, fNIRS optode locations on the head surface to an anatomical representation, including 3D images or meshes derived from the subject’s volumetric MRI, or an MRI atlas such as Talairach Atlas, MNI152, or Colin27. It is commonly called co-registration if the anatomical representation is the subject’s volumetric MRI. Registration allows for estimating the spatial location of brain activities or anatomical features and correlating with the signals probed by spatially distributed channels, assisting the interpretation of the observed brain activities in the context of brain anatomy. There are a number of algorithms to perform this procedure, such as rigid-body–based least-square fitting and iterative closest point (ICP)-based approaches.

### 5.25 Regularization

A range of algebraic operations that introduce a small perturbation on a tensor to alter its determinant. Consequently, regularization changes the condition number of a matrix. The range of applications of regularization is vast, e.g., perhaps for enabling or stabilizing matrix inversion, for altering the prominence of some dimensions of the tensor over others, and for increasing derivability, e.g., smoothing. By and large, the most popular regularization approach is Tikhonov’s which attacks the main diagonal isotropically and only has 1 parameter, but certainly, many other regularization approaches exist with different purposes. A popular use in fNIRS is to improve the stability and accuracy of the reconstruction algorithm by introducing additional constraints. It is often used in solving the ill-posed inverse problem in image reconstruction when the data being used for reconstruction is noisy and can help to reduce the effects of these sources of error on the final reconstructed image. Another form of regularization that has been used in fNIRS analysis is sparsity regularization, which adds a penalty term proportional to the L1 norm of the changes in hemoglobin concentration. This penalty term encourages the solution to be sparse, meaning that it has many zero or near-zero coefficients, which can help identify the brain regions that are most active.

### 5.26 Scattering

Scattering is the phenomenon by which there is a deflection of moving particles or radiation from their main direction by an interaction with other particles of matter in the medium through which they pass.

### 5.27 Sensitivity Matrix

The sensitivity matrix maps local absorption changes within the brain to variations of optical density changes measured by each channel. It is typically represented as a matrix with rows corresponding to the channels of the fNIRS system and columns corresponding to the voxels (or surface elements) in the head. The accuracy of the sensitivity matrix depends on various factors, such as the geometry and spacing of the fNIRS probes, the optical properties of the tissue being measured, and the assumptions made in the model used to estimate the matrix.

### 5.28 Shared Near-Infrared File Format (.snirf)

The shared near-infrared file format (SNIRF) is a standardized file format based on the hierarchical data format (HDF5) used for organizing, storing, and sharing near-infrared spectroscopy data and associated metadata. This format facilitates the exchange and analysis of fNIRS data across different hardware and software platforms. SNIRF is compatible with a variety of fNIRS analysis toolboxes and software platforms. It is designed to encapsulate various types of information, including metadata about the measurement, NIRS data, stimulus or trigger data, information about the probe set used, and any auxiliary data. The adoption of the SNIRF format helps streamline data sharing and collaboration within the fNIRS research community.

### 5.29 Short-Separation Channel

A short-separation channel is generated by placing a source and a detector at a short distance, in the case of the adult brain, this is typically ∼1  cm or less, in contrast to regular long-separation channels with ∼3  cm source-detector distances. At such distances, channels are mostly sensitive to systemic activity resulting from extra-cerebral tissues (i.e., scalp and skull). The signal from these channels can be used to regress out extra-cerebral contaminations from the long-separation fNIRS signal to isolate the cortical functional response.

### 5.30 Spectroscopy

Spectroscopy is a technique for analyzing and measuring the interaction between electromagnetic radiation and matter. In this process, a spectrometer records the intensity of radiation as a function of energy, frequency, or wavelength—the spectrum—after it interacts with or is emitted from a particular material being studied. Because these spectra depend on the absorption and emission properties of the material, and thus, on their physical and chemical structure, composition, and concentration, they can be used to classify materials.

### 5.31 Time-Domain Near-Infrared Spectroscopy

Time-domain near-infrared spectroscopy, in the context of fNIRS, refers to measuring the distribution of photon times of flight through tissue and fitting the resulting histogram to a theoretical model to calculate tissue optical properties, including absorption and scattering coefficients. TD-NIRS, and its Fourier domain analog swept frequency NIRS, permits gathering a richer data set than either single frequency-frequency domain NIRS or continuous wave NIRS. However, the cost of TD-NIRS hardware per detection channel tends to be higher.

### 5.32 Wavelength

Wavelength is the distance between corresponding points of a wave separated by one period. It is commonly denoted λ and is given by Eq. (12):

$$\lambda =\frac{u}{v},$$ (12)

where u is the wave speed and v is the frequency. In fNIRS, wavelength refers to the specific range of light wavelengths used to measure changes in chromophore concentration in the brain, which typically are between 650 and 900 nm.

## Data Availability

No new data were created or analyzed in this work. The consensus-based glossary is publicly available at https://openfnirs.org/standards/fnirs-glossary-project/.

## CRediT Taxonomy Statement

Conceptualization: KS, LKG, SMH, GAP, and MAYüc. Data curation—category documents: KS, LKG, SMH, GAP, and MAYüc. Data curation—writing original definitions: KS, LKG, SMH, GAP, MAYüc, SC, TDur, ACE, MF, JGev, RCM, FOE, VQ, FS, IT, AT, HW, AY, FK, CA, AB, BB, CBar, LB, CBul, DRB, MC, JMD, LD, TDre, AE, MME, LLE, AE, TRF, AF, SHF, JGem, CG, SMRG, EG, AFdCH, SMHH, DJ, ANKG, HK, AK, JKL, ML, RJM, DMAM, KLM, DM, MCM, RO, IP, PP, ARJ, VR, NAR, SSA, OSM, MS, SAU, MT, GV, MAYaq, CCY, and HZ. Funding acquisition: not applicable. Investigation: not applicable. Methodology: KS, LKG, SMH, GAP, and MAYüc. Project administration: KS, SMH, and MAYüc. Resources: not applicable. Software: SMH and MAYüc. Supervision: KS and MAYüc. Validation: not applicable. Visualization: FK: Fig. 1; FK: Fig. 3; FK: Fig. 4; FK: Fig. 5. Writing—original draft: KS and LKG. Writing—review and editing: KS, LKG, SMH, GAP, MAYüc, SC, TDur, ACE, MF, JGev, RCM, FOE, VQ, FS, IT, AT, HW, AGY, SAC, HD, QF, SF, YH, HN, HO, FK, CA, AB, BB, CBar, LB, CBul, DRB, MC, JMD, LD, TDre, AE, MME, LLE, AE, TRF, AF, SHF, JGem, CG, SMRG, EG, AFdCH, SMHH, DJ, ANKG, HK, AK, JKL, ML, RJM, DMAM, KLM, DM, MCM, RO, IP, PP, ARJ, VR, NAR, SSA, OSM, MS, SAU, MT, GV, MAYaq, CCY, and HZ.
